# Modifying the Frequency and Characteristics of Involuntary Autobiographical Memories

**DOI:** 10.1371/journal.pone.0089582

**Published:** 2014-04-09

**Authors:** Manila Vannucci, Iram Batool, Claudia Pelagatti, Giuliana Mazzoni

**Affiliations:** 1 Department of NEUROFARBA-Section of Psychology, University of Florence, Florence, Italy; 2 Department of Psychology, University of Hull, Hull, United Kingdom; Harvard Medical School, United States of America

## Abstract

Recent studies have shown that involuntary autobiographical memories (IAMs) can be elicited in the laboratory. Here we assessed whether the specific instructions given to participants can change the nature of the IAMs reported, in terms of both their frequency and their characteristics. People were either made or not made aware that the aim of the study was to examine IAMs. They reported mental contents either whenever they became aware of them or following a predetermined schedule. Both making people aware of the aim of the study and following a fixed schedule of interruptions increased significantly the number of IAMs reported. When aware of the aim of the study, participants reported more specific memories that had been retrieved and rehearsed more often in the past. These findings demonstrate that the number and characteristics of memories depend on the procedure used. Explanations of these effects and their implications for research on IAMs are discussed.

## Introduction

Involuntary autobiographical memories (IAMs) are spontaneously arising memories of personal events that come to mind with no deliberate attempt directed at their retrieval [1], [2]. Recent studies [3]–[6] have shown that IAMs can be elicited and experimentally investigated in the laboratory. In the present study we assessed whether the instructions given to participants can change the nature of memories reported. We show that changing specific details of the procedure strongly affects their retrieval, in terms of both their frequency and their phenomenological properties. Two variables have been manipulated in the present study, 1) whether people are made aware that the aim of the study is to examine IAMs and 2) whether they report their mental contents whenever they become aware of them or when requested to do so at random times set by a predetermined schedule.

Historically, the most common approach for studying IAMs has been the naturalistic diary method (e.g., [7]–[9]), in which individuals are asked to keep a diary of IAMs they experience in everyday life. These studies have shown that, when asked to report IAMs, people do so frequently, with routine daily occurrences of about 3–5 per day [7], [9], and that IAMs are at least as common (and presumably even more common) in daily life than are voluntary autobiographical memories [10]. They usually occur when one is engaged in undemanding activities that require little attention and concentration (e.g. during relaxation and routine activities) (e.g., [8], [11]). In most cases involuntary memories are reported to be elicited by identifiable cues that are generally related to prominent, possibly thematic, aspects of the remembered experiences (e.g., [7], [8], [12]).

The diary studies have also revealed that most IAMs refer to specific and mainly positive episodes (e.g., [13], but see [6]). Although diary studies provide many important basic findings, there are also intrinsic limitations related to this specific methodology, the inability to manipulate variables being the most obvious pitfall, as it limits the number of questions that can be addressed.

Two novel experimental methods have been successful in eliciting and measuring IAMs in the laboratory. They have simulated the conditions that in more naturalistic diary studies have shown to facilitate the production of IAMs, including using monotonous undemanding cognitive tasks. In a paradigm based on retrospective evaluations [3], participants were required to produce free associations to word cues (concrete nouns). At the end of the session, participants decided if a personal experience might have come to mind while giving these responses. Although the most participants provided a stream of semantic associations, participants also reported autobiographical memories on 86% of the trials.

In the other laboratory task [6], participants were asked to perform an undemanding vigilance task while being simultaneously exposed to irrelevant cue-phrases presented on the screen. Several involuntary memories were generated throughout the task, the majority triggered by the cues. When compared to voluntary memories obtained via a similar procedure [6], IAMs were more likely to be about specific past episodes and to be retrieved in response to negative cues. Retrieval time was almost twice as fast for IAMs than for voluntary memories.

In the word-association task developed by Ball [3], the participants were not provided with any information about involuntary memory retrieval until after they had provided all of their free associations. Thus, one might assume that they were not voluntarily retrieving autobiographical experiences to satisfy a demand characteristic of the experiment. Conversely, in the vigilance paradigm developed by Schlagman and Kvavilashvili [6] “participants were informed that some unrelated thoughts could be past memories that spontaneously “pop” to mind, and the nature of involuntary autobiographical memory was explained” ([6], p. 923).

As in diary studies, in this procedure people were informed that they were to report involuntary memories. Explicit instructions to report IAMs can have three unwanted effects. It can induce retrieval processes that are more similar to those of voluntary retrieval of autobiographical memories (hereinafter ABMs), in which case one should expect to find more memories compared to genuine involuntary retrieval, as in voluntary retrieval people typically report one memory per cue. A second possible effect is to create an overall priming of autobiographical contents, which would in general make ABMs more available. In this case too, one expects an increase in memories, and mainly of memories that are more accessible (e.g. those that have been previously rehearsed or reported). Third, instructions focusing on involuntary memories could also activate retrieval selection, setting the focus of attention on retrieval of ABMs or triggering a report bias that would limit the report to what people naively understand involuntary memories should be (e.g. specific personal events that are vivid and detailed). In all these cases, the retrieval and the nature of IAMs obtained might not be representative of naturally occurring IAMs, with the consequence that conclusions about the nature of IAMs reached with the Schlagman and Kvavilashvili [6] study might be incomplete or partially incorrect.

To assess this possibility, we compared involuntary memories reported in two conditions, one in which participants were told that the experimenters were interested in involuntary memories and another in which they were asked to report involuntary thoughts without any specific mention of memory. This was crossed with two other conditions, one in which participants were instructed to interrupt the task whenever a memory or a thought came to mind (depending on the condition) and one in which the task was interrupted by the experimenter according to a predetermined schedule. Not instructing participants to focus on involuntary memories and avoiding to mention of the word “memory” are supposed to enhance the chance to obtain memories that are truly involuntary. This should prevent priming of ABMs, and participants would less likely engage involuntary retrieval, or intentional selection of mental contents that they consider “memories”. These changes also made the task more similar to a typical mind-wandering task. In the present study we compared the number and characteristics of IAMs in the four conditions (see procedure below).

In both diary and laboratory studies on IAMs, participants are asked to report their memories using self-interruption. However, studies on mind wandering have shown that individuals routinely fail (at least temporarily) to notice that their minds have wandered, as they are only intermittently aware of their internal state (see for a review [14]). By contrast, when prompted by the experimenter, people can accurately report whether or not they are in a mind-wandering state. In response to queries about this procedure, they routinely indicate that they had been unaware of their mind wandering up until the time the probe was presented. Moreover, when participants classify mind-wandering episodes as unaware, their performance [15] and neurocognitive activity [16] systematically differ from when they report having been aware that they were mind wandering.

Similarly to what happens in mind-wandering tasks, in which people ‘zone out’ and are often unaware of the constant flux of mental contents, participants in our self-interruption condition might stop themselves only if they notice that they have a memory or thought. They might then omit reporting memories/thoughts on numerous occasions. However, if stopped they might become aware of having memories/thoughts at the moment or seconds earlier. We thus predicted a higher number of memories and thoughts in the experimenter-interruption conditions, because potential recovery from zoning out states would make participants realize they had been having thoughts/memories of which they were not aware of.

In the present study we used a modified version of the Schlagman and Kvavilashvili [6] paradigm, in which participants were asked to report whatever came spontaneously to their mind, including plans, generic thoughts, intentions for the future, past experiences, etc. Crucially, participants were not instructed to focus on involuntary memories and mention of the word “memory” was also avoided. These changes were intended to enhance the likelihood of obtaining memories that were truly involuntary. Not mentioning memory in the instructions should prevent priming of ABMs, and participants would less likely engage in voluntary retrieval, or intentional selection of mental contents that they consider “memories”. These changes also made the task more similar to a typical mind-wandering task.

In the new procedure, participants were instructed to report task-unrelated mental contents when these spontaneously popped into their mind, and to do so by interrupting the presentation of the stimuli and writing down a very short description of the mental content. They were told that the description, although short, should be sufficiently detailed to allow them to later identify what the mental content was. At the end of the presentation of all the stimuli, participants were asked to indicate which of the written mental contents referred to past events (i.e. memories) and which did not. With important differences, the method used in this study is similar to the two-step recording procedure extensively used in structured diary studies of IAMs (e.g., [7], [8], [17], [18]). In the original two-step method, participants make a preliminary record of the memory when the involuntary memory occurs, by recording keyword phrases in answer to a fixed set of questions listed in a small notebook. Step two involves filling out at a later time, self-chosen, a more extensive questionnaire about each memory. In our task, at step two participants identified the memory and elaborated on them. This might require more retrospection than the original two-step procedure.

The reason to have participants fill out the questionnaires at the end of the stimuli presentation was because a pilot study with the original Schlagman and Kvavilashvili [6] procedure (in which questionnaires were filled out after each single interruption) indicated that, although participants interrupted several times during the first half of the stimuli presentation, they stopped interrupting and reporting after a while and indicated that this was due to the need to fill out the questionnaire every single time. By shortening the immediate report we aimed at ensuring that participants reported memories throughout the vigilance task.

To summarize the whole design, we compared the effect on the frequency and characteristics of IAMs of two factors, a) Instruction type (2 levels, with *vs.* without mentioning IAMs) and b) Interruption type (2 levels, self-interruption *vs*. experimenter-interruption).

## Method

This research was approved by the Ethics Committee of the Department of Psychology, University of Hull, UK. Participants were asked to sign the informed consent form, which was part of the ethics application approved by the ethics committees. In it participants were told that the study examined the mental content of people during a vigilance task.

### Participants

Forty eight undergraduates from the University of Hull (29 females, age range 18–35, mean age 20.4) participated in the experiment. They were native English speakers, and had normal or corrected-to-normal vision. A preliminary informal interview assessed the presence of physical or mental problems and the consumption of medications. None of our participants had to be excluded on the basis of the results of the interview. Participants were randomly assigned to four experimental conditions, with 12 participants in each condition. Age difference among the four groups was not significant.

### Materials

#### Vigilance Task

The same vigilance task was used as in [6]. The task consisted of 800 trials, presented in a continuous fixed order, each remaining on the screen for 1.5 sec. In each trial an image was shown depicting either a pattern of black horizontal (non- target stimuli) or black vertical lines (target stimuli). Target stimuli appeared on 15 trials and were presented randomly every 40–60 trials, in order to ensure presentation at fairly long and irregular intervals. Each image showed also a word phrase (e.g. relaxing on a beach, supportive friend, see Appendix S2) in size 18 Arial in the middle of the image. The short sentences were taken from the standardized pool of 800 cues that had been rated for emotional valence by Schlagman and Kvavilashvili [6]. An approximately equal numbers of negative (*n = *267), neutral (*n* = 266), and positive (*n* = 267) phrases were used.

#### Questionnaire

Participants recorded details of each memory on the two-page questionnaire used by Schlagman and Kvavilashvili [6]. Instructions were exactly the same as those used by Schlagman and Kvavilashvili [6]. On the first page, participants wrote a brief description of the memory and rated the vividness of the memory on a 7-point scale (1 = very vague, almost no image at all; 7 = very vivid, almost like normal vision), indicated the trigger of the memory (their thoughts, an external trigger - in which case they had to mention which - or no trigger). On page 2, they rated on 5-point scales their overall level of concentration during the vigilance task (1 = not at all concentrating; 5 = fully concentrating), how unusual or common the remembered event was (1 = very common; 5 = very unusual), how often the memory had been thought of/rehearsed before (1 = never; 2 = once or twice; 3 = a few times; 4 = several times; 5 = many times), how pleasant or unpleasant the memory event was (1 = very unpleasant; 3 = neutral; 5 = very unpleasant), how pleasant or unpleasant the original event was (1 = very unpleasant; 3 = neutral; 5 = very pleasant). They were also asked whether the remembered event was general or specific, and indicated their age when the event occurred. Participants received instructions on how to identify a general and a specific event.

### Procedure

#### Design

This was a 2 (Instruction type: with *vs*. without mentioning IAMs)×2 (Interruption type: Self-interruption *vs*. Experimenter-interruption) design, with both factors manipulated between subjects.

Participants were tested individually. First they were asked to read information on the vigilance task explaining they were to detect randomly presented target stimuli (patterns of vertical lines) from a large number of non-target stimuli (patterns of horizontal lines). Each time a target stimulus was detected participants were to say “yes” out loud. They were told to ignore the words in the center of the pattern and that, due to the task being quite monotonous, they might find themselves thinking about other things, which was quite normal.

Instructions were the same in all conditions, except for the crucial differences that are mentioned below for each condition. The common part of the instructions is reported in Appendix S1.

Participants in the “IAM instructions/Self-interruption” condition received the original instructions as in Schlagman and Kvavilashvili [6]. In addition to the part in common to all conditions (see Appendix S1), in this condition they were told, “You may find that memories from your past come into your mind spontaneously without any deliberate attempt to retrieve them, in other words, a memory that simply ‘pops’ into your head without you trying to consciously remember anything. We are interested in studying these involuntary memories.” The nature of IAMs was explained. It was also specified that memories could be about specific or general events, from one's recent or remote past, and so forth. They were reminded that their main task was to respond by saying “yes” out loud each time they saw the target vertical lines, but if an involuntary autobiographical memory came to mind, then they were to click the mouse, which would stop the vigilance task and record their memory in one or two lines (i.e. a relatively short sentence). They were told that this initial brief description of the memory should be sufficient to remind them of the content of that specific memory at a later point in time. Participants were presented with their brief descriptions and asked to complete the two-page questionnaire for each memory only after all stimuli had been presented and all memories recorded.

Participants in the “IAM instructions/Experimenter-interruption” condition, received the same instruction as the first group but they were told that they would be interrupted during the performance and asked to report any involuntary memories that were going through their mind that they were aware of at the moment or/and just before the interruption. For each memory they were asked to briefly record the content as in the previous condition. The number of interruptions (*n* = 13) was established on the basis of the average number of interruptions (and memories) obtained in a pilot study with 15 participants using the standard self-interruption method. Interruptions were scheduled from trial 37 (1^st^ interruption) to trial 763 (13^th^ interruption) and were randomly spaced over a period of approximately 30 min.

Participants in the “No IAM instructions/Self-interruption” condition received the same instructions as those in the “IAM instructions/Self-interruption” condition, except that no mention was made of memories or past memories. Instead participants were asked to interrupt the presentation of the stimuli when task-unrelated mental contents (thoughts, plans, considerations, past events, images, etc.) “popped” into their mind. After all stimuli had been presented and all mental contents recorded, participants were informed about the nature of involuntary memories, saw their brief descriptions, asked to categorize the descriptions as involuntary memories or non-memory contents (that we called more generically thoughts), and asked to complete for each of the memories the two-page questionnaire.

Participants in the “No IAM instructions/Experimenter-interruption” condition received the same instructions as the previous group, but they were told that they would be interrupted during the performance and asked to report any involuntary mental contents that were going through their mind that they were aware of at the moment of or just before the interruption.

Each session lasted between 1½ and 2 hours, depending on the number of mental contents or involuntary memories generated.

## Results

All participants completed the vigilance task successfully. Classification of mental contents as memories or other mental contents was done relatively easily and quickly. Only on very rare occasions participants were uncertain whether the content was a memory or a more generic mental content.

Participants generated a total of 521 IAMs with a mean of 10.86 (*SD* = 9.02, range 0–37) per participant. Most importantly, out of 48 participants, only 2 participants did not report any involuntary memories throughout the session, one in the “No IAM instructions/Self-interruption” and one in “No IAM instructions/Experimenter-interruption”. Out of 521 IAMs, the majority (76%) were reported to have identifiable triggers. Out of these, 227 (57.3%) were reported to have been triggered by environmental cues and 169 (42.6%) by internal thoughts.

Descriptive data for all dependent variables are reported in Table 1. The total number of memories was 111 in the “IAM instructions/Self-interruption” condition; 215 in the “IAM instructions/Experimenter-interruption”; 83 (in addition to 124 thoughts) in “No IAM instructions/Self-interruption”; 112 (in addition to 206 thoughts) in “No IAM instructions/Experimenter-interruption”.

**Table 1:** Descriptive data** for all dependent measures.

	With mentioning IAMs		Without mentioning IAMs	
	SI^a^	EI^b^	SI^a^	EI^b^
**Memories**	9.25 (6.17)	17.92 (10.47)	6.92 (6.63)	9.33 (8.94)
	(1–22)	(7–37)	(0–26)	(0–29)
**Non-memories**	N/A	N/A	10.33 (5.30)	17.17 (9.56)
			(2–22)	(4–35)
**Vividness**	5.38 (1.09)	5.46 (.68)	5.29 (1.13)	5.45 (.60)
**Repeated before**	3.23 (1.15)	3.28 (.87)	2.44 (.81)	2.87 (.91)
**Specific (proportion)**	.74 (.18)	.75 (.20)	.55 (.34)	.64 (.23)
**Concentration**	3.75 (.91)	3.33 (.83)	3.13 (.81)	3.33 (.59)
**Unusual**	2.91 (.65)	3.17 (.61)	3.29 (.58)	3.15 (.58)
**Age of event**	18.08 (3.10)	17.50 (1.49)	19.95 (5.97)	17.64 (2.70)
**Pleasant. event**	3.27 (.86)	3.51 (.52)	3.53 (.39)	3.44 (.46)
**Pleasant. memory**	3.60 (.76)	3.49 (.48)	3.66 (.49)	3.57 (.48)

a SI = self-interrupted.

b EI = experimenter-interrupted.

**(first row: means and standard deviations; second row: min and max).

To assess the effect of type of instruction and type of interruptions on the total number of memories, the average number of IAMs per person was entered into a 2 (Instruction Type)×2 (Interruption Type) ANOVA. In the “IAM instructions” conditions, in which instructions mentioned IAMs, participants reported significantly more IAMs (*M* = 13.58) than participants in the “No IAM instructions” conditions (*M* = 8.12), (*F* (1,44) = 5.27, *p* = .027, eta squared = 0.09). The main effect of type of Interruption was also significant (*F* (1,44) = 5.43, *p* = .024, eta squared = 0.10). More IAMs were reported in the “Experimenter-interruption” (EI) (*M* = 13.62) than when in the “Self-interruption” (SI) (*M* = 8.08) conditions. The interaction was not significant (*F* (1,44) = 1.73, *p*>.19). Participants in the “Experimenter-interruption” condition also reported more mental contents (*M* = 17.17) than participants in the “Self-interruption” condition (*M* = 10.33) (*t* (22) = 2.17, *p* = .04, eta squared = .17). Recall that in the other “IAM instructions” conditions only memories were reported.

These results indicate that instructing participants about involuntary memories increased significantly the number of involuntary memories reported. A clear increase in memories was obtained also with scheduled interruptions. Having to report what they had in mind (memories or task-unrelated mental contents in general) at unexpected times apparently helped participants become aware of, and report, their mental contents.

To assess whether the two experimental manipulations affected the phenomenological quality of the involuntary memories retrieved, the mean ratings for each recorded memory characteristic were entered into 2×2 ANOVAs, with instruction type and interruption type as independent variables.

Participants in the “IAM instructions” conditions reported a higher proportion of specific memories (*F* (1,42) = 4.67, *p* = .036, eta squared  = .10), and a higher frequency of rehearsed memories (*F* (1,42) = 4.61, *p* = .038, eta squared = .10), compared to participants who received “No IAM instructions”. There was no difference between “Self-interruption” and “Experimenter-interruption” conditions in any memory characteristic, and no significant interaction.

## Discussion

The amount and type of involuntary memories reported depends strongly on the method used to elicit them. Informing participants that they had to report ‘involuntary memories’ (IAMs) during the vigilance task increased significantly the number of IAMs reported. These memories were also more specific and had been retrieved and rehearsed more than IAMs reported in the condition in which people were allowed to report any task-unrelated mental content.

In addition, more IAMs were reported when participants were interrupted by the experimenter compared to the self-interruption condition. However, the characteristics of the memories were not different between the two types of interruption. The lack of a significant interaction indicates that the influence of information about the type of task and about the type of interruption acted independently in triggering IAMs. In addition, the age of the memories, which typically dated back approximately 3 to 4 years, is consistent with previous results showing that involuntary memories refer to relatively recent periods in life (see [7], [8], [17]).

Several, and not mutually exclusive, explanations might be advanced for the effect of instructions and type of interruption. The greater number and different characteristics of memories obtained when instructions mentioned IAMs could be due to a priming effect, that might enhance the overall activation of autobiographical memories and help those already more active (e.g. more rehearsed) to pass the awareness threshold and ‘pop’ in mind (for other forms of chaining effects in IAMs, see [2], [3], [19]). The fact that memories reported when instructions focused on IAMs were indeed more rehearsed lends support to this interpretation. In addition, instructions focusing on involuntary memories might produce some form of selection at retrieval. This could be reflected in a report bias, in which only mental contents that reflect what people naively understand involuntary memories should be are reported. Memories are usually and naively conceived as referring to specific personal events, which are indeed the type of memories we found to be more frequent in the reports when the instructions explicitly stated that the aim was to study IAMs. Selection at retrieval could also be reflected in focusing attention during retrieval more narrowly just on memories, leaving out other mental contents. Conversely, in the “Self-interruption” group, in which ‘memory’ was not mentioned, attention would instead be spread across all mental contents that pop in mind, with the consequence that possibly some IAMs would go unnoticed. This would lead to a smaller number of IAMs in the “No IAM instructions” condition, as obtained here. In other words, when no memory instructions are given, people might end up omitting a number of IAMs that, if they paid attention, would be reported. Although the present data don't provide a definitive answer on whether this selection occurs at a pre-retrieval or post-retrieval level, the attentional explanation advanced for the “memory mentioning” effect receives some support also from the other result of this study, showing that a higher amount of IAMs is obtained when participants are unexpectedly interrupted by the experimenter (compared to the self-interruption condition).

In the “Experimenter-interruption” condition participants reported on average slightly more than one memory per interruption. While rather counterintuitive, this was predicted in light of previous results on ‘zoning out’ in mind-wandering tasks. Being unexpectedly interrupted and requested to report memories (in one condition) or mental contents (in the other) that come spontaneously to mind helps individuals become aware of mental contents that would otherwise go unnoticed [15], [16]. Recent theorizing about mind wandering suggests that meta-awareness (i.e. one's explicit knowledge of the current contents of thought), corresponds to an intermittent process whereby individuals only periodically notice the current contents of their mind. Direct comparisons between self-catching measures of the mind-wandering state (e.g. asking participants to press a response key every time they notice by themselves that they have been mind wandering) and probe-catching sampling (in which participants are intermittently queried whether or not they were mind wandering, and if they were, they are asked to indicate if they had been aware of this fact) have shown that individuals routinely mind wander without noticing it (zoning out) (see for a review and discussion [14]). In our case, random interruptions then make individuals aware of the mental contents at the time (and near the time) of the interruption, thus boosting the number of items reported. In the present data, this increase occurred not only for memories, but also for other mental contents (when instructions did not mention IAMs). Lack of awareness might also explain the relatively small number of thoughts and memories reported, in face of the large number of cues presented. Presenting so many cues might have a negative effect on the level of awareness as cues capture attention, leaving available fewer of the resources that are necessary in becoming aware of mental contents during the mind-wandering task.

An alternative explanation of the relatively greater frequency of reports in the experimenter interrupted condition is task demands (the interruption itself may have caused the participants to intentionally search and thereby possibly generate mental contents fitting the task). This would imply that intentional retrieval (or thought generation) would have occurred and the reports were not about unintentional mental contents or memories. However there is no reason why task demands should be different in the “Experimenter” and “Self-interruption” conditions. Future studies should assess more directly the role of task demands, and also examine the role of response bias.

The lower frequency of IAMs in the self-report condition could also be explained by higher cognitive demand due to greater monitoring of one's mind in order to notice and report IAMs. Previous studies have shown that IAMs are more frequent during undemanding tasks, and it is possible that self-interruption instructions could make the task more effortful when compared to the experimenter-interruption condition, where monitoring the content of consciousness occurs only when probed by the experimenter. If enhanced monitoring/awareness is the correct explanation for our data, then the greater number of mental contents and memories in the scheduled interruption condition suggests that diary studies, as well as studies using self-interruption procedures, in which people necessarily report only involuntary memories of which they are aware, have limited their investigation to involuntary memories that are sufficiently activated to spontaneously pass the awareness threshold. These can be memories with special qualities, in which case the theoretical explanations proposed so far on the nature and retrieval of IAMs might not extend to all involuntary memories.

In the present study, the random interruptions scheduled by the experimenter did not change the characteristics of the memories that were assessed, even if the number of memories reported increased. This might indicate that aware involuntary memories are not qualitatively different from unaware ones, and that passing the awareness threshold is a random event. However future studies should assess the effect on other characteristics of the memory, such as the degree of self-involvement in the event portrayed in the memory, or how the content is linked to self-relevant goals, etc., which represent crucial elements in autobiographical memory (see for example Conway's [20] model).

The lack of differences in characteristics suggest also that the increase in memories in this case should not be due to intentional selection or to reporting bias at retrieval. Rather, as in other forms of mind-wandering tasks, increase might be due to increased awareness of mental contents that without those interruptions would remain below threshold.

While all the explanations advanced so far are consistent with the present data, it will be the task of future studies to assess which explains them best. Future studies should also assess whether the differences obtained in the current experiment might also be due to differences in the nature of the retrieval process activated in the various conditions.

In the present work we also found a large inter-individual variability in the number of mental contents and memories reported during the vigilance task. This finding are probably inherent to the nature of the task (some people report substantially more while others substantially less), which would indicate that there are potentially important individual differences in cognitive and metacognitive processes between those who reported many mental contents and those who reported only a few. Future studies should investigate in a more systematic fashion the role of individual differences in metacognitive processes (monitoring and control) as well as personality variables (e.g. extroversion vs. introversion) on the tendency to report mental contents.

### Possible limitations of the current study

While we have so far referred to the memory reports obtained here as involuntary memories, in principle task-unrelated thoughts in mind-wandering tasks can be intentional, as they can be initiated in a goal directed fashion. For example, participants might intentionally make plans for the future or think about past events even just to relieve boredom. Thus, the memories identified using this methodology might not necessarily directly map on to the notion of involuntary memories (which are by definition unintentional).

While this is an important limitation, it does not apply just to the present study, but to all previous work on involuntary memories. The criticism is particularly true for those studies in which participants are directly and overtly asked to report involuntary memories, both in the lab [5], [6] and in diary studies [7]–[9]. However, in order to limit this potential problem, instructions in this and previous laboratory studies [6] clearly stated that participants were to report mental contents/memories that unintentionally, spontaneously, came to mind. Also, in the present study, the variations on the procedure (i.e., not mentioning memories at all for half of the participants) were devised with the aim to reduce the risk of reporting intentional (rather than unintentional) memories. This study also represents the first attempt to understand if different ‘involuntary’ memory procedures might produce memories of different quality.

In “No IAM instructions” conditions participants were asked to classify their mental contents only after completing the vigilance task, which might represent a limitation of this study. However, participants rarely had any difficulty in classifying what they reported as a memory or a more generic mental content. It is possible that in some (very rare) cases our data might have omitted some memories, or included some non-memories, but this would not change the pattern of results of this study. The results obtained in the present study are interesting and informative. However, future studies are necessary to assess whether our findings apply exclusively to involuntary memories, or characterize also voluntary autobiographical memories. This is an important point that has not been addressed here, but that might shed additional light to the actual nature of involuntary memories.

In the present study we examined a relatively small number of participants, and a replication of these results would be welcome. Nonetheless, we believe that the significant results will hold also in a larger sample as unpublished data from other labs reported a similar pattern of results.

### Strength of our results

Despite these limitations, our data clearly demonstrate, for the first time, that the procedure used to elicit IAMs strongly affects both the amount of memories obtained and their characteristics and that modifications in the procedure might change the results obtained about involuntary memories. This suggests that what is currently known about involuntary memories might still be very far from the final picture and probably important components of the processes involved in the retrieval of involuntary memories are still missing.

In any case, a firm point made by our result is to show the necessity of supplementing diary studies on involuntary memories with experimental work, if we want to reach an adequate understanding of how involuntary memories are retrieved. Very recent experimental work in this area (e.g. [4]) represents a very important step in that direction.

After having compared the four methods, we are in a better position to understand what their effect is. Is any of the four methods to prefer over the others? While we believe there are pros and cons with each method, the response to the question depends also on the aim of the study. The method in which participants are not informed that the aim of the study is to collect involuntary memories is in our opinion preferable to the one in which such directions are given, as these directions seem to change not only the likelihood of obtaining memories but also their characteristics. Self-interruptions seem to limit the output to memories that are over the awareness threshold, and thus should be used when the aim of the study is to examine the characteristics of these specific memories, or the variables that facilitate the report (output) of these memories. We believe, however, that the most interesting question about the retrieval of IAMs refers to understanding the factors that by activating existing information in autobiographical memory bring IAMs to an aware level. To this aim, in future studies one should compare Self and Experimenter-interruption conditions when participants are not aware that the aim is on IAMs and manipulate variables that are likely to increase the activation of memory representations, such as priming, which has already been shown to affect involuntary memory report [19], [21]. In one study [22], we have shown, for example, that both explicit and implicit priming affects the rate of IAMs. In this way, one can compare memories that are still below the level of awareness and reach awareness only when people are asked to focus their attention on spontaneous mental activity, with memory representations that have received sufficient activation by external cues to be above the aware level. Much still needs to be done to understand how IAMs are retrieved, and the results future studies can help understand the extent to which factors that typically modulate retrieval in voluntary memories also affect involuntary retrieval.

## Conclusions

This study shows that both instructions and procedure affect the rate and in part the characteristics of involuntary memories, a result that suggests that it is possible that the findings of previous studies might be limited to some types of involuntary memories and not others. In addition, these data hint at the possibility that involuntary memories, as products of the mental activity of a mind-wandering task, are one component of a rather constant flux of mental contents that can pass or not pass the awareness threshold and thus capture attention and be reported.

## Supporting Information

Appendix S1 **Instructions received by all participants.**
(DOC)

Appendix S2 **A subset of cue words used in the experiment.**
(DOC)
