# Supplementary material for: Modifying the Frequency and Characteristics of Involuntary Autobiographical Memories
Source: PLoS One. 2014 Apr 9;9(4):e89582. doi: 10.1371/journal.pone.0089582 (PMC3981656; doi:10.1371/journal.pone.0089582)
Supplement: Appendix S1 — Instructions received by all participants. (DOC) [file pone.0089582.s001.doc]

**Appendix S1**

The aim of this task is to study people’s ability to concentrate on a relatively monotonous and repetitive task. In order to study your concentration you will be shown a large number of cards on a computer screen one by one. The cards will show patterned lines. Most of the time a pattern of horizontal lines will be shown and you should ignore these. Occasionally you will see a pattern of vertical lines, these are the targets which you should detect. Each time you detect vertical lines on a card presented on the screen you should respond by saying “yes” out loud and the computer will automatically record your response and time taken to respond to this target. In addition to seeing horizontal and occasionally vertical lines you will also see some words in the middle of the cards. You do not need to do anything with these words as we are looking at your ability to keep track of patterns, just continue to react to the target lines when they appear. In another condition, participants will have to concentrate on the words and ignore the lines. Therefore, the overall aim of the study is to investigate which stimuli are better for concentration (the words or patterns).

Although this seems like a simple task it is sometimes quite difficult to concentrate on a monotonous task and your thoughts may drift away from the task. You may find yourself unintentionally thinking about other things that are unrelated to the task, for example, you may find yourself begin to think about your plans for this evening, your current situation, daydreams or events that have happened to you in the past. It is very normal to have such thoughts, and in addition to your concentration abilities we are also interested in some of these unrelated thoughts that you may experience.
