# Supplementary material for: Modifying the Frequency and Characteristics of Involuntary Autobiographical Memories
Source: PLoS One. 2014 Apr 9;9(4):e89582. doi: 10.1371/journal.pone.0089582 (PMC3981656; doi:10.1371/journal.pone.0089582)
Supplement: Appendix S2 — A subset of cue words used in the experiment. (DOC) [file pone.0089582.s002.doc]

**Appendix S2**

| Music concert |
| --- |
| Christmas presents |
| Disney World |
| Family pet |
| Clear blue sky |
| A glass of wine |
| Stars at night |
| Relaxing on a beach |
| Refreshing drink |
| Beautiful view |
| Dinner with friends |
